# Ameliorative Effect of Spinach on Non-Alcoholic Fatty Liver Disease Induced in Rats by a High-Fat Diet

**DOI:** 10.3390/ijms20071662

**Published:** 2019-04-03

**Authors:** Laura Inés Elvira-Torales, Gala Martín-Pozuelo, Rocío González-Barrio, Inmaculada Navarro-González, Francisco-José Pallarés, Marina Santaella, Javier García-Alonso, Ángel Sevilla, María Jesús Periago-Castón

**Affiliations:** 1 Department of Food Technology, Food Science and Nutrition, Faculty of Veterinary Sciences, Regional Campus of International Excellence “Campus Mare Nostrum”, Biomedical Research Institute of Murcia (IMIB-Arrixaca-UMU), University Clinical Hospital “Virgen de la Arrixaca”, University of Murcia, Espinardo, 30071 Murcia, Spain; galamartin@um.es (G.M.-P.); rgbarrio@um.es (R.G.-B.); inmaculada.navarro@um.es (I.N.-G.); marinasp@um.es (M.S.); fjgarcia@um.es (J.G.-A.); 2 Department of Food Engineering, Tierra Blanca Superior Technological Institute, 95180 Tierra Blanca, Veracruz, Mexico; 3 Department of Anatomy and Comparative Pathological Anatomy, Faculty of Veterinary Sciences, Regional Campus of International Excellence “Campus Mare Nostrum”, University of Murcia, Espinardo, 30071 Murcia, Spain; pallares@um.es; 4 Anchormen, Pedro de Medinalaan 11, 1086 XK Amsterdam, The Netherlands; asevilla@um.es

**Keywords:** spinach, carotenoids, steatosis, gene expression, metabolomic, lipid metabolism

## Abstract

The purpose of this work was to evaluate the effect of dietary carotenoids from spinach on the inflammation and oxidative stress biomarkers, liver lipid profile, and liver transcriptomic and metabolomics profiles in Sprague–Dawley rats with steatosis induced by a high-fat diet. Two concentrations of spinach powder (2.5 and 5%) were used in two types of diet: high-fat (H) and standard (N). Although rats fed diet H showed an accumulation of fat in hepatocytes, they did not show differences in the values of adiponectin, tumor necrosis factor alpha (TNF-α), and oxygen radical absorption (ORAC) in plasma or of isoprostanes in urine compared with animals fed diet N. The consumption of spinach and the accumulation of α and β carotenes and lutein in the liver was inversely correlated with serum total cholesterol and glucose and the content of hepatic cholesterol, increasing monounsaturated fatty acids (MUFA), polyunsaturated fatty acids (PUFA) and reducing cholesterol in the livers of rats fed diet H and spinach. In addition, changes in the expression of genes related to the fatty liver condition occurred, and the expression of genes involved in the metabolism of fatty acids and cholesterol increased, mainly through the overexpression of peroxisome proliferator activated receptors (PPARs). Related to liver metabolites, animals fed with diet H showed hypoaminoacidemia, mainly for the glucogenic aminoacids. Although no changes were observed in inflammation and oxidative stress biomarkers, the consumption of spinach modulated the lipid metabolism in liver, which must be taken into consideration during the dietary treatment of steatosis.

## 1. Introduction

Non-alcoholic fatty liver disease (NAFLD) is the liver disease which is disseminated most widely around the world due to genetic, dietary, and lifestyle factors [1]. Day et al. [2] suggested a two-stage development of NAFLD: firstly, an accumulation of triglycerides and free fatty acids in the hepatocytes, and secondly, lipid peroxidation, mitochondrial dysfunction, and liver inflammation. These processes result in an increase in fatty acid synthesis and a decline in β-oxidation and in the exportation of triglycerides from the liver as very low-density lipoprotein (VLDL) [3].

For green leafy vegetables, such as spinach, a number of functional properties have been found regarding their nutrients and bioactive compounds, such as antioxidant, anti-inflammatory, anti-proliferative, anti-obesity, hypoglycemic, and hypolipidemic activity [4]. Among the commonly consumed leafy vegetables, spinach can be considered a source of bioactive compounds such as phenolic compounds and carotenoids. In relation to phenolic compounds, spinach only contributes approximately 0.8 mg of gallic acid equivalents/day/person, due to the low daily intake [4]. However, spinach is considered one of the richest plant sources of carotenoids, contributing to the intake of lutein, zeaxanthin, and carotene. Carotenoids can contribute positively to liver health [5,6], and their consumption has been associated with decreased fat accumulation in the liver in patients with NAFLD [7], being effective in the prevention and treatment of this liver pathology [6]. However, the mechanisms have not yet been elucidated, and further investigations must be conducted. In animal studies, it has been observed that lycopene reduces fat accumulation and inflammation of the liver through the activation of the antioxidant and anti-inflammatory response, increased transport of cholesterol and fatty acids, improvement of β-oxidation, and regulation of mRNA translation [8,9]. Lutein (the most abundant carotenoid in spinach) reduced cholesterol, malondialdehyde (MDA), and tumor necrosis factor alpha (TNF-α) levels in the liver of guinea pigs when administered in hypercholesterolemic diets [10], but this effect depends on the form in which lutein is administrated [11]. The supplementation of the diet with astaxanthin could reduce the expression of the peroxisome proliferator-activated receptor gamma (PPARG) and DNA damage inducible-transcript 3 (*CHOP-10*) genes, thereby diminishing hepatic lipid transport and fatty acid synthesis and avoiding the development of hepatic steatosis [12]. The administration of β-cryptoxanthin significantly reduced steatosis in mice by decreasing oxidative stress and the inflammatory response by inhibiting the expression of related genes [13]. Although the influence of several pure carotenoids on liver health has been reported, information about the preventive effect of dietary carotenoids on NAFLD is still rare.

Because spinach is a natural source of carotenoids, its utilization in a whole-food intervention approach provides multiple nutrients with a broad range of biological activities, creating the potential for complementary, additive, or synergistic activities that are lacking when supplementation involves only a single nutrient. Moreover, given that the prevention of a disease at an early stage is the principle of dietary treatment, the purpose of this work was to evaluate whether supplementation of the diet with spinach, as a dietary source of carotenoids, has an effect on biomarkers of the steatosis of Sprague–Dawley rats fed a high-fat diet. To achieve this general objective, we have evaluated the changes in the plasmatic parameters, inflammation and oxidative stress markers, liver lipid content, and transcriptomic and metabolomic profiles of rats after supplementation of their feed with spinach powder.

## 2. Results

### 2.1. Feed Composition, Weight Gain and Volume of Feed Consumption

Table 1 shows the proximate composition, energy values, total phenolics and carotenoids of the six experimental diets—NC (standard diet), N2.5 (standard diet + 2.5% spinach), N5 (standard diet + 5% spinach), HC (high-fat diet), H2.5 (high-fat diet + 2.5% spinach) and H5 (high-fat diet + 5% spinach)—which were administered to the rats for five weeks. The feed of H groups provided a higher content of protein and fat, resulting in a mean energetic value of around 450 kcal/100 g. In addition, this diet showed a lower proportion of total dietary fiber (TDF) and total phenolic compounds (TPC) than N diets due to the low content of unrefined agricultural commodities. In general, the incorporation of spinach in both diets did not led to significant differences in the composition of the diets, only regarding carotenoid intake. The administration of 5% spinach in the feed provided a mean content of 9.1 μg of total carotenoids/g of feed, while the supplementation with 2.5% spinach gave 3.1 μg of carotenoids/g of feed. According to the carotenoid profile of spinach, lutein and α-carotene were the predominant carotenoids in the feed, followed by β-carotene, whereas neoxanthin and violaxanthin were not detected in the feed.

Table 2 shows the consumption of food and water, changes in body and liver weight, feces and urine excretion, and daily carotenoid intake during the intervention period. The initial mean body weights did not exhibit significant differences among the six experimental groups. In contrast, at the end of the experimental period, the body weight increases and liver weight differed significantly between the animals fed the N diets and those fed the H diet, due to the higher caloric value of the latter diet. In addition, the liver weight was significantly lower in H2.5 and H5 than in the H group. Excreted feces values were significantly higher in N groups in comparison with H groups, which could be explained by the daily intake of TDF. Despite the higher content of TFD in H2.5 and H5 diets, no differences were observed in the amount of excreted feces with HC. According to the proportion of spinach and the content of carotenoids in the feed, the consumption of these compounds was significantly higher (*p* < 0.05) in the groups that received 5% spinach (55.5 and 53.2 μg/day for N5 and H5, respectively) than in groups N2.5 and H2.5 (Table 2). Differences in the daily intake of total TPC were also observed between the N and H diet.

### 2.2. Histopathological Examination and Biochemical Parameters

**Figure 1:**
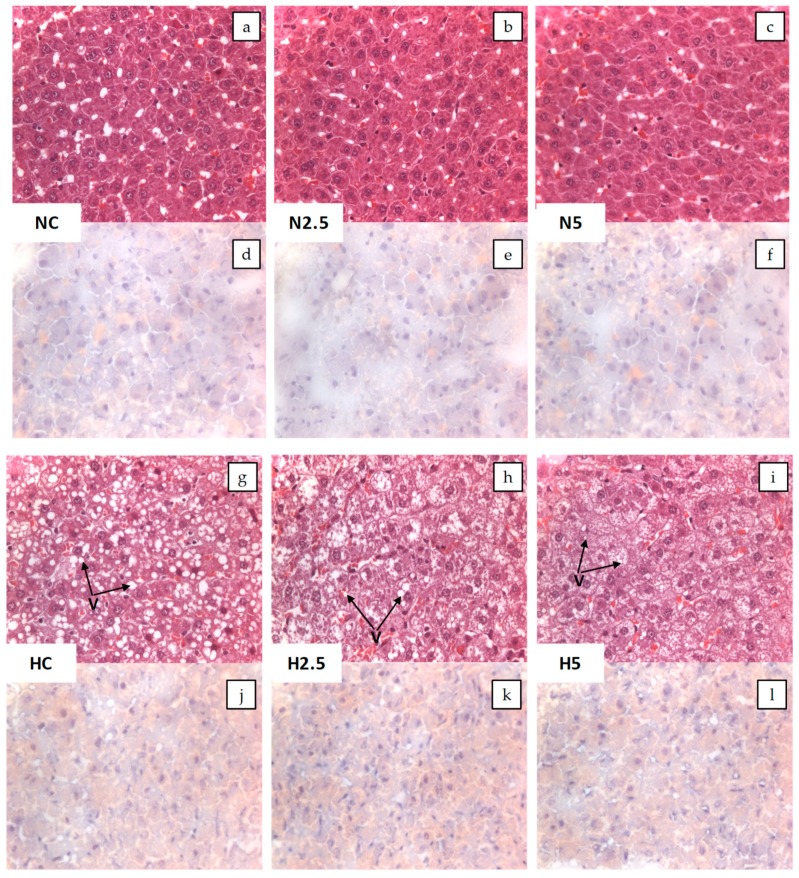
Microscopic photographs of liver tissue. Microscopic images with H&E (**a**–**c** and **g**–**i**) and Sudan III (**d**–**f** and **j**–**l**) visualized by light microscopy (×40) for the control and experimental groups. Arrows show the vacuolar degeneration of the hepatocyte (V).

Considering the anatomical and pathological examination (Figure 1), the presence of steatosis in rats of the H groups can be observed in both the macroscopic and microscopic images. Macroscopically, the liver was enlarged, yellow, and greasy (pictures not shown). Microscopically, the hepatocytes contained small and large vesicles due to the abnormal accumulation of lipids, particularly triglycerides. The accumulation of fat was confirmed using Sudan III, which stains triglycerides and other intracellular lipid droplets, providing an orange color. According to the number of vacuoles, the steatosis was classified as grade 3, with 50–75% of the hepatocytes showing vacuolar degeneration. However, after the consumption of spinach (H2.5 and H5 rats), the vacuoles were slightly smaller in comparison with those of animals that had received the high fat diet.

In addition, the infiltration of mononuclear cells and the degeneration and necrosis of hepatocytes were evaluated to determine the inflammation level. Only a low grade of inflammation was detected (grade 1), with less than 20% of the examined area affected. The steatosis was confirmed by the analysis of the plasmatic transaminase enzymes alanine aminotransferase (ALT) and aspartate aminotransferase (AST), whose activities showed an increase at the end of the experimental period (Table 3). It can be observed that rats fed diet N showed a normal histopathological liver (Figure 1).

The biochemical parameters of the plasma (levels of glucose, protein and hepatic enzymes) are shown in Table 3. The glucose concentration did not differ significantly among the N groups, but did among the H groups, being significantly reduced in rats of group H5 (153.6 mg/dL), in contrast to the total protein level, which remained unchanged at the end of the study. As mentioned above, rats of the H groups showed a significantly higher level of hepatic enzymes than animals of N groups, indicating disturbances in the liver functionality.

**Figure 2:**
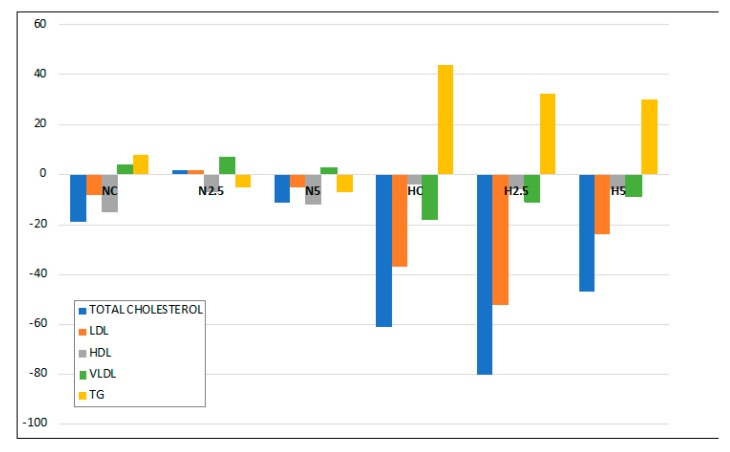
Changes in lipid parameters measured in plasma at the beginning and at the end of the intervention period of 5-weeks for the six experimental groups. LDL: low-density lipoprotein; HDL: high-density lipoprotein; VLDL: very low-density lipoprotein; TG: triglycerides.

Regarding the plasma lipid levels, Figure 2 represents the changes between initial and final values. No significant changes were observed for total cholesterol, low-density lipoprotein (LDL), high-density lipoprotein (HDL), VLDL, or triglycerides (TG) in groups NC, N2.5, and N5 during the intervention period. In contrast, important changes were observed between the initial and final parameters for the H groups, showing a significant drop in total cholesterol, LDL and VLDL and a significant increase in plasmatic TG (Figure 2). This trend was due to the steatosis, since the metabolism of lipoprotein is altered, significantly increasing the content of plasmatic TG. The consumption of spinach only leads to a significant reduction in final cholesterol (108 in HC, 91 in H2.5 and 75 in H5), and triglycerides (123 in HC, 102 in H2.5 and 103 in H5), showing a hipocholestrelomic effect (data not shown). Other parameters measured were the inflammation and oxidative stress biomarkers. For oxygen radical absorption capacity (ORAC) in plasma and for urinary isoprostanes, there were no significant differences between the initial (data not shown) and final values or among the different conditions (diet and spinach supplementation, Table 3). The levels of adiponectin and TNF-α did not change either; no significant difference was found according to the diet or the consumption of spinach.

### 2.3. Content of Carotenoids, Total Fat, Fatty Acids and Cholesterol in the Liver

The bioavailability of carotenoids was measured considering their accumulation in the liver. For groups N5, H2.5, and H5, the total carotenoid accumulation in the liver was 0.20, 0.29 and 1.45 μg/g, respectively. The carotenoid with the highest concentration in the liver was β-carotene, whereas the lowest concentration was observed for lutein. In N2.5, NC, and HC rats, no carotenoids were detected (Table 4).

**Figure 3:**
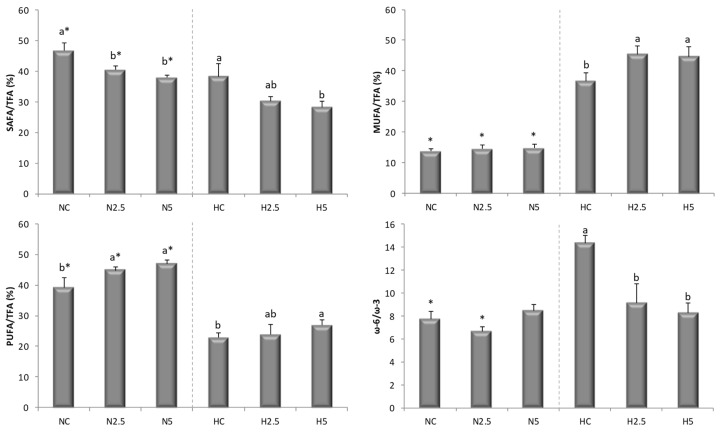
Saturated fatty acid (SAFA)/total fatty acid (TFA), monounsaturated fatty acid (MUFA)/TFA, polyunsaturated fatty acid (PUFA)/TFA, and ω-6/ω-3 ratios in the liver of rats of the six experimental groups. ^a,b^ Different letters show significant statistical differences (*p* < 0.05) among groups fed the standard diet (NC, N2.5, N5) or the high-fat diet (HC, H2.5, H5) after performing a one-way ANOVA. * Significant statistical difference (*p* < 0.05), after carrying out a two-sample *t* test, between the members of the NC-HC, N2.5-H2.5, and N5-H5 pairings. Result are expressed as mean ± SD.

The accumulation of total fat in the liver of animals of the H groups was five times greater than in the N groups (mean values: 25.35% *vs*. 5.51%, data not shown), and the consumption of spinach had no effect on the liver total fat content. Nevertheless, spinach consumption and the accumulation of carotenoids in the liver appeared to have an effect on the accumulation of cholesterol, since there were significant reductions in hepatic cholesterol in groups H2.5 and H5, which reached healthier values with respect to the control (HC) (Table 5). In addition, a significant reduction of cholesterol was detected in the liver of animals of group N5. The analysis of total fatty acids in the liver showed differences in the quantities of specific fatty acids between N and H groups (Table 5). A higher proportion of monounsaturated fatty acids (MUFA) was observed in animals fed the H diet compared to those fed the N diet, which showed a high proportion of saturated (SAFA) and polyunsaturated fatty acids (PUFA). The addition of spinach positively influenced the fatty acid profile of the liver, significantly reducing the contents of SAFA as well as significantly increasing the content of PUFA in N and H groups. Also, an increase of MUFA was observed, but only in rats fed H diets (Figure 3); although n-3 (linolenic acid (ALA), eicosapentaenoic acid (EPA), docosahexaenoic (DHA)) and n-6 (linoleic acid (LA), eicosadienoic acid (EDA), and arachidonic acid (AA)) increased in animals fed the H diet supplemented with spinach (Table 5), the n-6/n-3 ratio decreased significantly (Figure 3).

### 2.4. Gene Expression Related to NAFLD

Twenty-seven differentially expressed genes were selected from the fatty liver array (Table 6), according to the criteria indicated above. All genes with differential expression showed an overexpression of the mRNA; none was down-expressed. In general terms, the group in which the most major changes in the transcriptome were observed was N5, showing changes in genes related to β-oxidation (3 genes), cholesterol and other lipid transport and metabolism (14 genes), and the inflammatory response and apoptosis (6 genes). For animals of the HC and H5 group, 5 and 11 genes were overexpressed, respectively. According to these results, a fat diet led to changes in gene expression, but higher changes were observed in animals that took in spinach and accumulated carotenoids in their livers.

### 2.5. Metabolites in Liver

**Figure 4:**
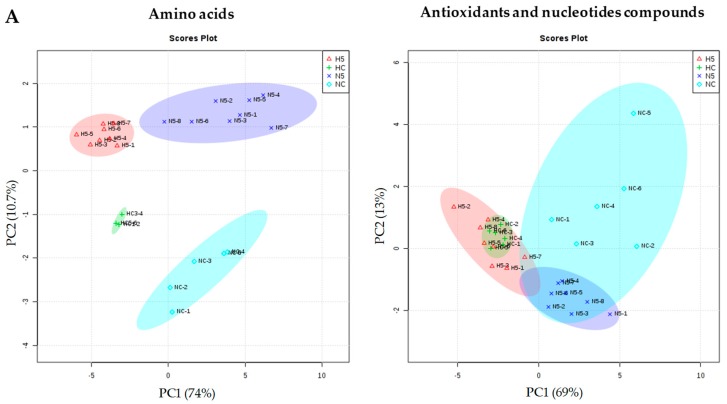

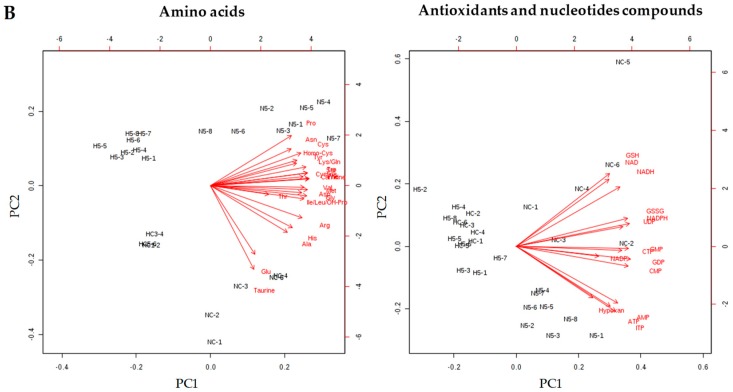
(**A**) PCA scores and (**B**) eigenvector plots for the amino acids, antioxidants, and nucleotides compounds, according to the metabolites found to differ significantly (ANOVA *p* < 0.05) among the different diets: group NC: standard diet, N5: standard diet + 5% spinach, HC: high fat diet and H5: high fat diet + 5% spinach. Confidence regions are marked with different ellipses.

Regarding the liver metabolites, the principal component analysis (PCA) shows a great influence of the type of diet (standard or high fat) on the metabolites (represented by PC1), accounting for 74% of the total variance in the amino acids and 69% of the total variance in the antioxidant and nucleotide components (Figure 4). The spinach supplementation of the diet (represented by PC2) accounted for 10.7% of the total variance in the amino acids and 13% for the other components analyzed. Hence, the total variance explained jointly by PC1 and PC2 was 84% for the amino acids and more than 82% for the other components. In global terms, a standard diet had a positive influence on all the metabolites; however, the effect of spinach (PC2) mainly altered redox molecules such as _L_-glutathione (GSH), l-glutathione oxidised form (GSSG), l-homocysteine (Homo-Cys), nicotinamide adenine dinucleotide oxidised form (NAD), and nicotinamide adenine dinucleotide reduced form (NADH), showing a modulation of the redox responses, which could be associated with the antioxidant capacity of the carotenoids. In addition to the above, a change in nucleotides (adenosine monophosphate (AMP), adenosine triphosphate (ATP), and inosine triphosphate (ITP)) and some amino acids (l-proline (Pro), _L_-asparagine (Asn), l-cysteine (Cys), l-arginine (Arg), l-histidine (His), l-alanine (Ala), l-glutamic acid (Glu), and taurine) occurred, which indicates an effect on the redox metabolism and some other key points in the rat metabolism.

**Figure 5:**
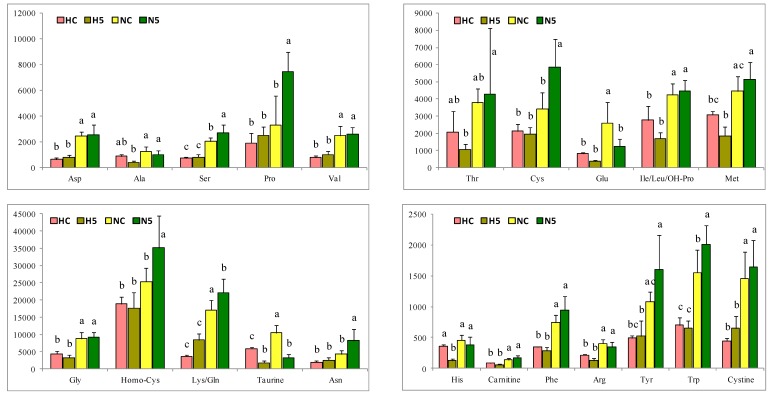
Amino acid content in the liver of rats of the four experimental groups (HC: high fat diet, H5: high fat diet + 5% spinach, NC: standard diet and N5: standard diet + 5% spinach). The bar height indicates the mean value of each feed condition and the error bar indicates the standard deviation. ^a–c^ Different letters indicate significant statistical differences (*p* < 0.05).

**Figure 6:**
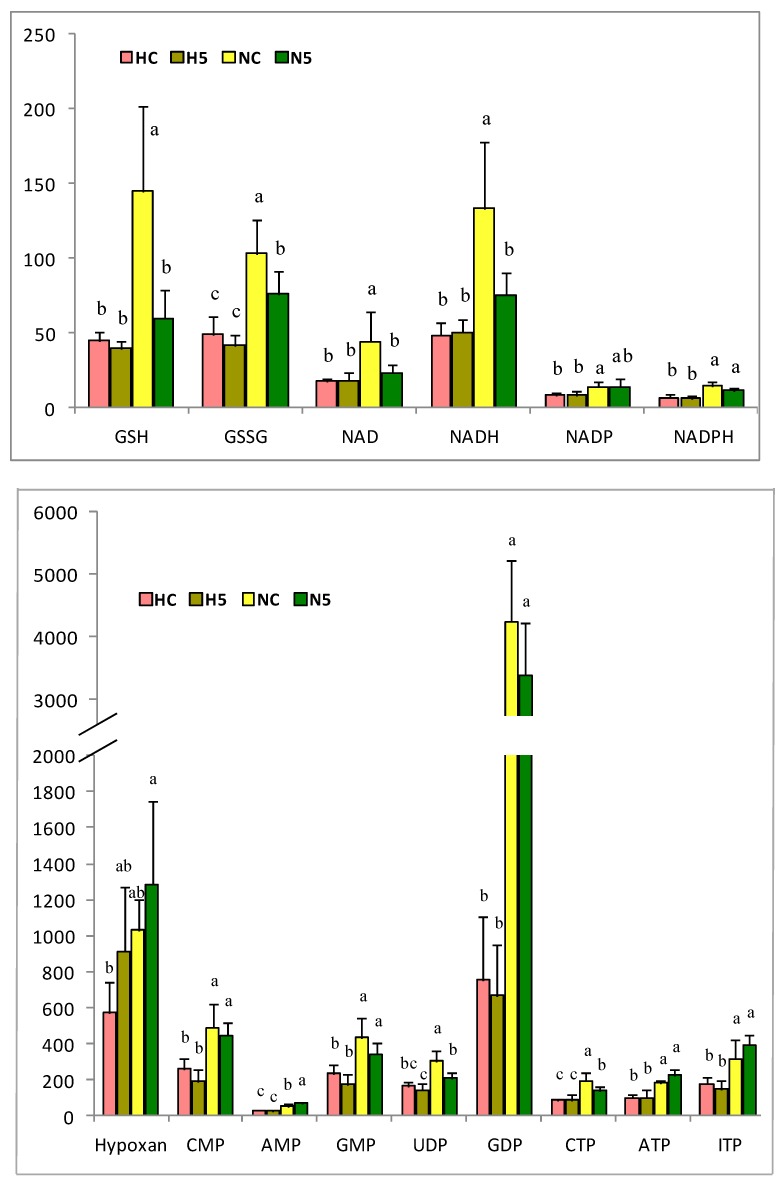
Antioxidant and nucleotide compounds in the liver of rats of the four experimental groups (HC: high fat diet, H5: high fat diet + 5% spinach, NC: standard diet and N5: standard diet + 5% spinach). The bar height indicates the mean value of each feed condition and the error bar indicates the standard deviation. ^a–c^ Different letters indicate significant statistical differences (*p* < 0.05). GSH: l-glutathione; GSSG: l-glutathione oxidized form; NAD: nicotinamide adenine dinucleotide; NADH: nicotinamide adenine dinucleotide reduced form; NADP: nicotinamide adenine dinucleotide phosphate; NADPH: nicotinamide adenine dinucleotide phosphate reduced form; CMP: cytidine monophosphate; AMP: adenosine monophosphate; GMP: guanosine monophosphate; UDP: uridine diphosphate; GDO: guanosine diphosphate; CTP: cytidine triphosphate; ATP: adenosine triphosphate; ITP: inosine triphosphate.

In general, a significant reduction in the amino acid content was observed in the animals of HC and H5 groups, in comparison with the NC and N5 groups. When the diet of the healthy animals was supplemented with spinach, the concentrations of some amino acids increased significantly (l-serine (Ser), Pro, Cys, Asn, l-lysine/_L_-glutamine (Lys/Gln), Homo-Cys, l-tryptophan (Trp)), whereas those of Glu and taurine declined (Figure 5). The antioxidant and nucleotide compounds also decreased under the fatty diet (Figure 6). However, in rats fed diet N, the consumption of spinach significantly reduced the concentrations of redox compounds (GSH, GSSG, NAD, NADH) and of some nucleotides (uridine diphosphate (UDP) and cytidine triphosphate (CTP)). The H diet and the consumption of spinach influenced the GSH/GSSG ratio of the liver, decreasing in rats fed H diets in comparison with the NC group, and, similarly, a significant reduction was observed in the N5 group (Table 7). The ratios of NAD/NADH and nicotinamide adenine dinucleotide phosphate oxidised form/nicotinamide adenine dinucleotide phosphate reduced form (NADP/NADPH) remained unchanged among the four experimental groups (Table 7).

## 3. Discussion

### 3.1. Carotenoid Supplementation and Biomarkers of Stetaosis

In the present study, the intake of high fat provoked steatosis in rats in the H groups, as described by different authors [9,14,15]. Animals fed the high-fat diet showed a significant increase in the activities of hepatic enzymes and microvesicular steatosis. However, this hepatic disturbance was not severe, because there were no clear symptoms of lipotoxicity and steatohepatitis, as revealed by the inflammation and oxidative stress biomarkers. Although the consumption of spinach had no clear effect in the inflammation and stress biomarkers, we observed in the histological examination a reduction of the lipid size of vacuoles and a significant reduction in the total weight of the liver and plasmatic glucose levels in H2.5 and H5 groups. This effect of carotenoids on glucose metabolism has been reported in previous in vivo studies using dietary carotenoids [16,17], showing a beneficial effect on steatosis features by decreasing insulin resistance. Although some plasmatic parameters remained within the normal range [18,19], from the beginning to the end of the experiment, it was expected that the most significant changes would occur in the lipid profile of the plasma due to the alteration of lipid metabolism by the hepatic steatosis, increasing total cholesterol and TG. In contrast, a decrease in total cholesterol and lipoprotein LDL and VLDL could be explained by a decline in the synthesis of lipoprotein in the liver, so that lipids accumulated in the hepatocytes instead of being liberated into the peripheral circulation [20], whereas TG increased in plasma. It is remarkable that the consumption of spinach significantly reduced the proportion of plasmatic TG, indicating a role in lipid metabolism.

The role of carotenoids in the regulation of specific functions and in the prevention of disease is determined by their bioavailability. Carotenoids are absorbed through the mucosa of the small intestine by passive diffusion, lodging inside the chylomicrons, which are rich in triglycerides, due to their lipophilic nature, and are transported in the lymph to the liver [21]. Later, carotenoids are transported by the LDL and are incorporated into the inner body of the lipoproteins, whereas xanthophylls become attached to their surface; for this reason, xanthophylls are transported at a greater rate to other organs [22]. The accumulation of total carotenoids in the liver (carotenes and lutein) was significantly correlated with the amount of spinach provided and the type of diet, since the fat content of the feed facilitated the absorption of carotenoids, as has been mentioned in other studies [9]; for this reason, in rats in group N2.5, carotenoids were not detected in the liver.

Although carotenoids were absorbed and accumulated in the liver of rats of N5, H2.5 and H5 groups, these antioxidants did not appear to have a significant effect on the biomarkers of oxidative stress and inflammation in plasma. Ko et al. [23] reported that the oxidative stress caused by hyperlipidemia can be partly prevented by the antioxidant activities of spinach administrated at 5%, together with a fat-rich diet. However, these authors described a decline in thiobarbituric acid reactive substances (TBARs) in the liver, but not in plasma. Other researchers have reported that several dietary carotenoids—such as lycopene from tomato juice administered ad libitum, lutein (100 mg/g of diet), zeaxanthin (0.25 mg/g), and astaxanthin (0.2 mg/g)—can diminish the oxidative stress and/or levels of biomarkers of cellular inflammation in rats [6,9,10,16]. These concentrations are higher than those assayed in this study. Hence, to evaluate the beneficial effect of spinach on these biomarkers, a higher concentration in the diet could be required. Although no changes were observed for plasma adiponectin and TNF-α, the modulation of the inflammatory response by carotenoids depends on different factors, such as specific compounds and their concentrations, but also on the level of oxidative stress [24]. Carotenoids have been used to reduce the inflammatory response through effects on the transcription system of nuclear factor-κB (NF-κB). However, the results are contradictory: in different cell cultures, lycopene was found to repress NF-κB, while β-carotene stimulated it [25]. Changes in the genes related to the inflammatory response were studied for the NC, H5, and N5 groups, and will be discussed later.

The depletion of PUFA in the H groups indicates a decline in fatty acid oxidation and triglyceride release from the liver, with a consequent increase in triglyceride synthesis that may have contributed significantly to the triglyceride accumulation in hepatocytes [26]. The consumption of spinach led to significant changes in the liver fatty acid profile, which became healthier, with increases in the proportions of MUFA and PUFA and decreases in SAFA and the n-6/n-3 ratio. These changes could be considered beneficial with respect to the inhibition of stress-related kinases and apoptosis [27], since it has been reported that MUFA decreases intracellular lipid levels and the markers of inflammation, increasing fatty acid oxidation and triglycerides synthesis [28]. Moreover, this behavior suggests that the accumulation of carotenoids stimulated the conversion of fatty acids into long-chain unsaturated products, yielding α-linolenic acid (C18:3 n-3, ALA) and eicosadienoic acid (C20:2 n-6, EDA), as described by Bell et al. [29] for the flesh of salmon. In our study, when the high-fat diet was supplemented with spinach, the MUFA and PUFA concentrations increased significantly, reducing the SAFA contribution (Figure 2). The most plausible explanation is a higher activity of both desaturases and elongase enzymes, as described by other authors [30]. These changes reduce or inhibit the de novo synthesis of fatty acids and activate their β-oxidation by the stimulation of MUFA and PUFA. The decrease in the n-6/n-3 ratio indicates an increase in the concentration of n-3 fatty acids, which limits the storage of triglycerides in the liver, reducing the risk of the development of NAFLD [31]. In addition, n-3 PUFAs are precursors of anti-inflammatory eicosanoids, as opposed to n-6 PUFAs, which produce pro-inflammatory eicosanoids [32]. When carotenoids were present in the liver, the concentrations of the ALA (C18:3 n-3 α), EDA, EPA (C20:5 n-3), and DHA (C22:6 n-3) fatty acids increased, improving the liver fatty acid profile in health terms.

In addition, carotenoids also influenced the reduction of the cholesterol level in the liver of rats with steatosis, as has been described in the scientific literature for different carotenoids. Kim et al. [10] reported a significant reduction in the percentage distribution of free cholesterol in the liver of rats after the administration of 0.1 g/100 g of lutein. Nicolle et al. [33] reported that supplementation of the diet with 0.25% cholesterol and 20% lyophilized carrots significantly reduced the cholesterol levels in plasma and in the liver of C57BL/6J mice. Qiu et al. [34] fed Sprague–Dawley rats with a high-fat diet supplemented with 50 mg of lutein/kg body weight/day and found a reduction in the cholesterol content in the liver, which correlated with the concentration of lutein in the diet. In other animal studies involving a high-fat diet supplemented with 20 mg of lycopene/kg body weight/day or 30 mg of astaxanthin/kg body weight/day, the administration of these carotenoids reduced the concentration of cholesterol in the liver to levels similar to the control [35,36]. In general, these authors used carotenoid-rich plant material or pure compounds at a dose 4–8 times higher than that used in our experiment. Although we used a lower dose, liver cholesterol decreased in rats with steatosis after the intake of spinach, as well in healthy animals when taking in around 50 μg/day of total carotenoids. Different mechanisms have been proposed for the reduction of cholesterol accumulation in the liver; one is the inhibition of 3-hydroxy-3-methylglutaryl-CoA reductase (HMGCR) activity, as has been described for other carotenoids such as lycopene [37] and β-carotene [38].

### 3.2. Bioactivity of Diets Supplemented with Spinach: Modulation of Gene Expression

Although the available evidence regarding the potential use of non-provitamin A carotenoids in the prevention and treatment of NAFLD suggests that these compounds are effective in decreasing lipid accumulation, insulin resistance, oxidative stress, and inflammation in hepatic tissue, more complex pathways related to gene expression have not been elucidated completely [6]. In this research, we observed that the intake of a fatty diet and spinach led to changes in the expression of genes related to fatty liver disease. In the N5 group—the healthy animals that consumed spinach—there was an over-expression of a great number of genes in comparison with the control group (NC). The over-expression of genes related to β-oxidation, Acyl-Coenzyme A dehydrogenase, long-chain (*ACADL*), Carnitine palmitoyltransferase 2 (*CPT2*), and peroxisome proliferator activated receptor alpha (*PPARA*) was observed. *ACADL* encodes a dehydrogenase enzyme that catalyzes the initial step in each cycle of fatty acid β-oxidation; this is one of a class of enzymes that are important due to their role in the metabolism of fatty acids present in the diet [39]. In addition, *CPT2*, together with carnitine palmitoyltransferase 1A, liver (*CPT1A)*, oxidizes long-chain fatty acids in the mitochondria, which allows the linkage of acyl-CoA derivatives to a polar molecule of carnitine, resulting in the formation of acylcarnitine molecules that are transported into the mitochondria [40]. In this study, only *CPT2* was overexpressed, but in a previous study, we reported that the intake of lycopene from tomato juice increased the mRNA abundance of *CPT1A*, together with the carnitine content, in the liver of rats with steatosis [8,9].

The peroxisome proliferator activated receptors (PPARs) consist of three members—PPARA, peroxisome proliferator-activated receptor gamma (PPARG), and peroxisome proliferator-activated receptor delta (PPARD)—which form obligate heterodimers with the retinoid X receptor (RXR). Carotenoids and their metabolites are activators of these receptors [25,41], which are involved in the transcriptional regulation of several pathways of lipid metabolism. *PPARA* is expressed in the liver and has been considered to exert a critical role in the prevention of fat-related oxidative stress, inflammation and NAFLD [42]. *PPARD* and *PPARG* were also over-expressed in N5 rats, showing the effect of carotenoids from spinach on the activation of other types of PPAR receptors, expressed in other tissues [43]. In addition, the acyl-CoA synthetase medium-chain family member 3 gene (*ACSM3)*, which encodes a protein that participates in the synthesis of medium-chain fatty acids, was over-expressed, showing an enhancement of lipid metabolism. The activity of this protein leads to a higher influx of fatty acids into the mitochondria, and in particular facilitates the oxidation of medium-chain fatty acids (from C4 to C11), since small- and medium-chain acyl-CoA derivatives have the ability to cross the inner mitochondrial membrane by diffusion [40]. For these reasons, rats fed with spinach (groups N5 and H5) had a lower SAFA/TFA ratio in the liver (Table 6 and Figure 2).

Related to cholesterol transport and metabolism, the over-expression of apolipoproteins (*APOA1*, *APOB*, and *APOE*) and membrane receptors such as low-density lipoprotein receptor (*LDLR*) and *ABCG1* was observed, which could have led to an increase in the activity of proteins involved in cholesterol transport. *ABCG1* was over-expressed, increasing the efflux of cholesterol and phospholipids to lipid-poor apolipoproteins. This transporter is a major regulator of cellular cholesterol and phospholipid homeostasis, since cholesterol taken up by the liver can be recycled back through the ABCG1 pathway, being secreted into bile—as either free cholesterol or bile acids—or assembled into lipoprotein particles that are secreted back into the circulation [44]. The overexpression of *ABCG1* was accompanied by the overexpression of the mRNA of apolipoproteins A, B, and E, and also that of the LDL receptor, which indicates an increase in lipoprotein metabolism. We also found an over-expression of the mRNA of genes involved in the repression of the synthesis of different lipids, such as nuclear receptor subfamily 1, group H, member 3 (*NR1H3*), nuclear receptor subfamily 1, group H, member 4 (*NR1H4*), CCHC-type zinc finger, nucleic acid binding protein (*CNBP*) and sterol regulatory element binding transcription factor 2 (*SREBF2*), which are responsible for the inhibition of the synthesis of cholesterol and bile acids. LXR (NR1H3) is involved in liver lipid metabolism [45] and exhibits a homeostatic effect at the transcriptional level. This receptor regulates the synthesis of lipids via SREBF2 and the excretion of bile acids, by activating cytochrome P450, family 7, subfamily A, polypeptide 1 (*CYP7A1*), thereby reaching a balance in the content of hepatic cholesterol [46]. This effect has been described after the consumption of other carotenoids, such as lycopene from tomato, by rats with NAFLD induced by a fatty diet [47].

Additionally, the genes involved in the inflammatory response increased their activity, as demonstrated by overexpression of the genes suppressor of cytokine signaling 3 (*SOCS3*), caspase 3 (*CASP3*), and mitogen-activated protein kinase 8 (*MAPK8*), which participate in anti-inflammatory and apoptotic processes. Other carotenoids, such as lycopene, can prevent oxidative stress in hepatocytes and also modulate the transcriptome response of genes related to apoptosis and regulation of the cell cycle, particularly by the over-expression of the tumor-suppressor protein (TP53), which acts as a major defense against cancer through the differential activation of target genes [48]. However, it is important to highlight that the adiponectin receptor 1 (*ADIPOR1*) and nuclear factor (*NFKB1*) genes were over-expressed, which also could be considered beneficial for the liver. Adiponectin has been shown to have cytoprotective properties, improving both hepatic and peripheral insulin sensitivity and preventing steatosis, inflammation, and necrosis, whereas the inhibition of *NFKB1* induces non-alcoholic steatohepatitis (NASH) and hepatocellular carcinoma (HCC) by sensitizing hepatocytes to undergo spontaneous apoptosis [49]. Also, in the N5 group, the over-expression of *IL1B*, considered an inflammatory cytokine, was observed, but no negative effect was detected in the rats.

In the HC group (rats with steatosis induced by the fatty diet), genes such as *ABCG1* and *LPL* were over-expressed. These genes encode the proteins that facilitate the extracellular transport of lipids and the hydrolysis of TG in free fatty acids, respectively. The gene expression of *LPL* is higher in obese subjects with NAFLD than in subjects without NAFLD, suggesting that free fatty acids released by the lipolysis of circulating triglycerides also contribute to hepatocellular fatty acid accumulation and steatosis [50]. In addition, there was an increase in the expression of the transcription factor *PPARG*; this is responsible for increasing insulin sensitivity and for promoting the entry of fatty acids and storage of triglycerides in adipocytes [51]. Additionally, the *IL1B* and *TNFR* (*FAS*) genes were overexpressed, revealing inflammation in the adipocytes—a result of the accumulation of fat. These changes are in concordance with the disturbance of lipid metabolism associated with NAFLD and with lipotoxicity [51].

Unlike the HC group, the H5 group showed some similarities with the N5 group, which indicates that spinach consumption had a positive effect on the amelioration of the fatty liver condition. For this reason, a positive modulation of the genes involved in the β-oxidation of fatty acids was observed, associated with the overexpression of *CPT1A*, *CPT2*, and the nuclear factor *PPARA*. With regard to cholesterol transport and metabolism, the *APOE* gene, which encodes lipoproteins, was also over-expressed, but to a lesser extent in comparison to the N5 group. Hence, spinach intake promoted the expression of these genes, so that liver cholesterol declined due to its efflux from the liver through the more abundant VLDL and LDL apoliproteins. The cholesterol catabolic activity was enhanced by the over-expression of the cytochrome P450, family 2, subfamily E, polypeptide 1 (*CYP2E1*) and *CYP7A1* genes, responsible for the augmented synthesis of bile acids through the overexpression of *NR1H4*. *FXR* (*NR1H4*) stimulated the expression of *CYP7A1* in H5 rats, keeping the synthesis of bile acids active and thus leading to a decline in hepatic cholesterol [45]. PPARG is required for adipocyte differentiation and for the maintenance of differentiated adipocyte, and is considered as an adipogenic factor, since it is an activator of fatty acid synthesis and storage. However, it plays divergent roles in the metabolism, and these effects in the metabolism are controversial. Thus, *PPARG*’s activation by thiazolidinediones, the most investigated synthetic agonist, results in the increased production of adipokines, including adiponectin, which enhances hepatic fatty acid oxidation. In addition, it also promotes fat storage in adipocytes and decreases adipose tissue lipolysis, thereby decreasing the concentration of fatty acids stored in the liver. Moreover, its activation also exhibits an anti-inflammatory role. This effect is associated with the increase of insulin sensitivity, improving the resistance of insulin associated with the steatosis and metabolic syndrome [52]. In this study, we do not analyze the resistance of insulin, but the supplementation of spinach and the accumulation of carotenoids in the liver (H5 group) exhibited a significant effect on the reduction of plasmatic glucose, total cholesterol and TG, as well as cholesterol in the liver. Finally, the *ADIPOR1* and *CASP3* genes were over-expressed, as described above for rats of the N5 group.

### 3.3. Changes in Liver Metabolites

The amino acids analyzed in the metabolomic study had lower concentrations in rats fed the high-fat diet because of the high caloric content of this diet, which produced a hypoaminoacidemic effect [53], mainly for the glucogenic amino acids, confirming the results obtained in studies performed with hyperlipidemic diets. The concentrations of the intermediaries of the redox process (Met and taurine) were decreased in the H5 group, which may be due to the spinach intake and the antioxidant effect of the accumulated carotenoids in the liver [54]. A plausible explanation is that the level of endogenous antioxidant molecules was reduced, and hence the GSH/GSSG ratio showed this same reduction. Although different authors have considered that a high level of Homo-Cys in plasma is related to NAFLD, being a critical factor in the pathogenesis groups [55,56], in this study, the liver content of Homo-Cys was highest in the animals of the N5 group; however, this cannot be considered a negative effect, since the animals of this group also had a high liver content of methionine. The GSH/GSSG ratio was altered by the treatments, type of diet, and supplementation with spinach, being lowered by the antioxidant effect of spinach. A plausible explanation could be that the high antioxidant content in the diet determined a lower in vivo response for maintaining the redox balance, and hence the use of reduced glutathione significantly decreased. This effect agrees with the findings of other authors who used tomato extract, lycopene, and astaxanthin and obtained a reduction in the redox ratio [57,58].

## 4. Materials and Methods

### 4.1. Spinach and Preparation of Diets

Spinach (*Spinacia oleracea*) was obtained from a local supermarket as a fresh-cut product. The edible part was boiled for 10 minutes to remove oxalic acid, the water was discarded, and then the cooked spinach was lyophilized and ground. The powdered samples were stored at 4 °C until their use. The total content of carotenoids in spinach powder was determined by HPLC and was 1750 µg/g, showing the following amounts of the individual compounds: 228 µg of neoxanthin/g, 292 µg of violaxanthin/g, 944 µg of lutein/g, 46 µg of α-carotene/g, and 225 µg of β-carotenene/g. The spinach-enriched diets were prepared by mixing the pulverized pellets of the standard diet (Teklad Global 14% Protein Rodent Maintenance Diet TD-2014; Harland Laboratories, Indianapolis, IN, USA) or the high-fat diet (Atherogenic rodent diet TD-02028; Harland Laboratories) with 2.5% and 5% freeze-dried spinach powder. Water was added to each of the mixtures until a mass was formed which was not sticky. The pellets were then prepared using a pastry bag and dried in a tray dryer at 60 °C for 21 h. Dried pellets with spinach were packed in polythene bags and stored in the refrigerator until they were used.

### 4.2. Animals and Experimental Design

**Figure 7:**
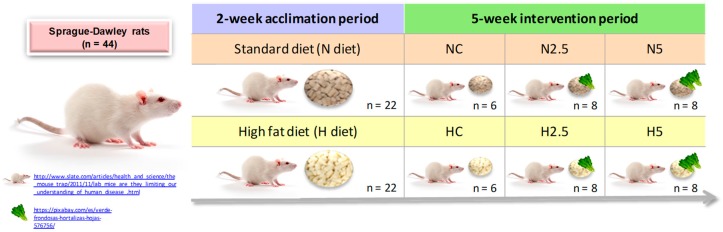
Experimental design of the study.

The experimental protocol of this work was approved by the Ethical Committee of Animal Experimentation of the University of Murcia and by the General Directorate of Livestock and Fisheries of the C.A.R.M. (No. A1320140701, permitted on 23 July 2014). Based on preliminary studies, the sample size (*n*) was estimated by comparing two proportions using the following formula [59]:

$$n=\frac{{\left[z_{1-\alpha /2}\sqrt{\{2\bar{p}(1-\bar{p}}\}+z_{1-\beta }\sqrt{\left\{P_{A}\left(1-P_{A}\right)+P_{B}\left(1-P_{B}\right)\right\}}\right]}^{2}}{\delta^{2}}$$

where $z_{1-\alpha /2}$ is the *z*α value corresponding to the desired risk, $z_{1-\beta }$ is the *z*β value corresponding to the statistical power, $P_{A}$ is the value of the proportion in the control group, $P_{B}$ is the value of the proportion in the group of the treatment, $\bar{p}$ is the average of the two proportions $P_{A}$ and $P_{B}$, and $\delta^{2}$ is the $P_{A}$ − $P_{B}$. The sample size obtained was adjusted for 10% attrition. It was estimated using the following formula [60]:

$$Sample\;adjusted\;to\;the\;attrition=n\left(\frac{1}{1-R}\right)$$

where *n* is the number of subjects without attrition and *R* is the expected proportion of attrition. Forty-four male adult Sprague-awley rats (8 weeks of age) were grouped into two groups (*n* = 22) according to their diet: standard diet (diet N) or a high-fat diet (diet H). These diets were administered for two weeks; after this time, the animals were classified into six experimental groups. There were two control groups (*n* = 6 rats/group), a standard diet (NC) and a high fat diet (HC), and four experimental groups (*n* = 8 rats/group): N5 (standard diet + 5% spinach), N2.5 (standard diet + 2.5% spinach), H5 (high fat diet + 5% spinach), and H2.5 (high fat diet + 2.5% spinach). The experimental period was five weeks (Figure 7). Body weight was registered weekly and feed intake and urinary and fecal excretions were recorded in the initial, middle, and final parts of the experimental period, using metabolic cages for data collection. At the end of this period, the rats were sacrificed and the different biological samples (plasma, feces, urine, and liver) were obtained. All the samples were stored at −80 °C until the analytical processes were performed.

### 4.3. Histopathological Examination

Histological examinations were carried out in the Pathological Anatomy Service of the Veterinary Hospital of the University of Murcia. Samples of the liver from each animal were taken and each sample was divided into two parts. One was fixed in formalin and paraffin-embedded, and 4-μm-thick sections were obtained and stained with hematoxylin–eosin. The other was snap-frozen in 2-methylbutane cooled in liquid nitrogen and stored at −70 °C until use. Frozen 5-μm-thick sections were cut with a cryostat at −20 °C and stained with Sudan III for lipids detection. The liver sections were examined (without knowledge of their experimental group) and given an estimated score for the severity of interstitial hepatitis: 0 = no microscopic lesions; 1 = mild interstitial hepatitis; 2 = moderate multifocal interstitial hepatitis; 3 = severe multifocal interstitial hepatitis. The degree of steatosis was evaluated as follows: 0 = no vacuolar degeneration; 1 = less than 25% of hepatocytes affected; 2 = 25–50% of hepatocytes affected; 3 = 50–75% of hepatocytes affected; 4 = more than 75% of hepatocytes affected.

### 4.4. Plasma Biochemical Parameters

Glucose, total protein, insulin, total cholesterol, fractions of HDL-cholesterol, LDL-cholesterol, and VLDL-cholesterol, TG, and activity of the enzymes AST and ALT were analyzed in plasma samples, using an automatic analyzer (AU 600 Olympus Life, Hamburg, Germany) in the Veterinary Hospital of the University of Murcia.

### 4.5. Determination of Biomarkers of Inflammation and Oxidative Stress

The TNF-α and adiponectin levels in plasma were determined using a commercial ELISA kit (Single Analyte ElisarrayTM kits for Rat; QIAGEN, SA Biosciences, Frederick, MD, USA). The plasma antioxidant capacity, expressed as mmol of Trolox equivalents (TE)/L, was determined by the ORAC technique, using a multimodal microplate reader (Synergy HT BioTek, Winooski, VT, USA) [61]. Urinary excretion of 15-F2t-isoprostane (8-epi-PGF2α) was determined with an ELISA kit (OxySelectTm-epi-PGF2α Elisa Kit, Cell Biolabs), and the creatinine concentration was used to normalize the constituents [62]. These parameters were measured at the beginning and end of the intervention period.

### 4.6. Analysis of Carotenoids in the Spinach and Liver

The analysis of carotenoids was carried out according to the procedure described previously by our research group [9]. Carotenoids were extracted twice with tetrahydrofuran/methanol (1/1, *v*/*v*) containing 0.1% butylhydroxytoluene. The combined extracts were brought to dryness in a rotary evaporator and the residues were suspended in 5 mL of (TBME/MeOH). The carotenoids were analyzed by HPLC (Agilent 1200, Waldbronn, Germany), with a C_30_ column (250 × 4.6 mm, 5 μm i.d.) (Trentec, Gerlingen, Germany) at 17 °C, using a TBME (A) and MeOH (B) mobile phase with a flow rate of 1 mL/min. The gradient used began with 2% A in B, reaching 35% A at 35 min, 60% A at 45 min, and 60% A at 56 min, before returning to the initial conditions for 4 min before the next injection. Detection of the carotenoids was carried out using a diode array detector (DAD) system at 450 nm. Standard curves were prepared using reference standards for quantification.

### 4.7. Analysis of Total Dietary Fiber (TDF) and Total Phenolic Compounds (TPC) in Feed

The TDF was determined according to the AOAC procedure (985.29) (1990) described by Prosky et al. [63]. The TPC were determined, using Folin Ciocalteu’s Phenol reagent, according to Hirawan et al. [64].

### 4.8. Analysis of Lipids in the Liver

The contents of total fat, fatty acids, and cholesterol were determined in the rat livers. Total fat was analyzed by the Soxhlet method [65], using ethyl ether as solvent. Fatty acids and cholesterol were analyzed using a Sigma Aldrich Lipid Extraction Kit (MAK174, St. Louis, MO, USA) and Cholesterol Extraction Kit (MAK175), respectively, following the extraction method of Folch et al. [66] and the procedures described by the manufacturer. The quantification of fatty acids and cholesterol was performed on a GC (Agilent GC 7890A, Palo Alto, CA, USA) equipped with a flame ionization detector (FID), as reported by Martin-Pozuelo [9].

### 4.9. Study of the Expression of Genes involved in Fatty Liver Disease

Liver samples were used for the analysis of gene expression, following the procedure previously described by Martin-Pozuelo et al. [9]. Real-time PCR analyses were carried out according to the manufacturer’s instructions, using a 96-well PCR array for the evaluation of fatty liver disease genes (PARN-157ZD-24, Qiagen, SABiosciences, Frederick, MD, USA). Relative gene expression was determined according to the comparative *C*t method. The gene expression was only investigated in rats of the control groups (NC and HC) and in those that had ingested high levels of spinach (N5 and H5).

### 4.10. Analysis of Liver Metabolites by HPLC-MS

The extraction of metabolites was based on the work described previously by our research group [8]. After extraction, the samples were injected into an Agilent 1200 series HPLC instrument (Agilent Technologies, California, USA) coupled to an Agilent 6120 single quadrupole mass spectrometer with an orthogonal ESI source. In order to avoid potential degradation of metabolites, the samples were prepared shortly before chromatographic analysis. The analysis of metabolites was only carried out with the liver of animals belonging to the control groups (NC and HC) and the groups with high intake of spinach (N5 and H5)

### 4.11. Statistical Analysis

All analytical determinations were performed in triplicate and the data were expressed as the mean ± standard deviation of the results obtained. In all cases, the normality and equality of variances were tested. A one-way ANOVA with repeated measures was applied, with a post-hoc test to determine differences among the means of all the analytical determinations: Tukey’s test or the Games–Howell test according to the case. For the data of food and drink intake, weight gain and excreta, and cholesterol and fatty acids in the liver, the two-sample Student’s *t* test was performed to compare groups N and H. In addition, for each biochemical parameter and biomarker of inflammation or oxidative stress analyzed, a paired Student’s *t* test was performed to compare the values at the beginning and end of the experiment. The level of significance was *p* < 0.05. For the analysis of liver metabolites, the concentrations were normalized according to the weight of the tissues and a two-way ANOVA was carried out, followed by a post-hoc Tukey HSD test; its familywise error rate was corrected using the Benjamini–Hochberg false discovery rate (FDR) with a 5% proportion of false discovery [67]. In addition, a principal componentaAnalysis was applied. The statistical analysis was carried out with the IBM Statistical Package for the Social Sciences (SPSS), version 24.0. The significance of the relative gene expression, taking NC as the control group, was determined with Partek® Genomics Suite 6.6., considering the genes with differential expression to be those satisfying the following criteria: a fold change > 2 or < −2 and *p* < 0.05.

## 5. Conclusions

We can summarize that, in rats with steatosis, the consumption of spinach and the accumulation of carotenoids in the liver reduced the content of SAFA, the ω-6/ω-3 fatty acid ratio and cholesterol, and increased MUFA and PUFA, as well as modified the expression of genes related to the fatty liver condition, increasing the abundance of proteins involved in the metabolism of fatty acids and cholesterol, mainly through the overexpression of PPARs. Moreover, the supplementation of spinach improved the expression of genes related to inflammatory response. Therefore, based on the results obtained, spinach can be considered as part of a dietary strategy in the control and treatment of NAFLD as a natural and safe source of carotenoids. However, this research underlines the need for further investigation to elucidate the effect on the content of specific enzymes and to determine the doses that could regulate lipid metabolism in the treatment of this pathology.

## Figures and Tables

**Table 1:** Proximate composition, energy values and total phenolic compounds in the experimental diets ^1^.

Parameters	NC	N2.5	N5	HC	H2.5	H5
Protein (g/100 g)	14.5	15.0	15.5	17.3	17.7	18.1
Fat (g/100 g)	4.0	4.0	4.0	21.2	20.8	20.4
Total dietary fiber (TDF) (g/100 g)	21.2	21.3	22.6	6.9	7.4	8.0
Carbohydrate (g/100 g)	55.6	55.0	53.2	51.1	50.6	49.9
Starch (g/100 g)	34.4	33.7	30.5	44.2	43.1	41.9
Ash (g/100 g)	4.7	4.7	4.7	3.5	3.5	3.6
Energetic value (kcal/100 g)	316.3	316.0	310.8	464.4	460.1	455.4
Calories from protein (%)	18.3	19.0	19.9	14.9	15.4	15.9
Calories from fat (%)	11.4	11.4	11.7	41.1	40.6	40.3
Calories from carbohydrate (%)	70.3	69.6	68.4	44.0	44.0	43.8
Total phenolics (TPC) (mg GAE/100 g)	188.4	192.2	196.1	20.4	21.2	22.4

^1^ Values are expressed as mean.

**Table 2:** Food and drink intake, excreted feces and urine, and carotenoid intake of the six experimental groups in the 5-week intervention period ^1^.

Parameters	NC	N2.5	N5	HC	H2.5	H5
Initial body weight (g)	371.9 ± 33.9	380.4 ± 15.2	383.3 ± 19.9	377.8 ± 20.5	372.3 ± 28.2	387.6 ± 5.7
Final body weight (g)	459.4 ± 43.7	451.4 ± 22.5	442.0 ± 16.6	500.1 ± 37.3 ^b^	475.1 ± 34.4 ^b^	552.5 ± 29.9 ^a^
Body weight increase (g)	87.55 ± 18.85 *	71.02 ± 12.71 *	68.21 ± 9.32 *	122.28 ± 21.83 ^ab^	102.85 ± 22.09 ^b^	154.5 ± 29.2 ^a^
Liver weight (g)	15.32 ± 2.16 *	13.92 ± 1.80 *	13.65 ± 1.90 *	25.36 ± 3.06 ^a^	21.44 ± 2.95 ^b^	19.31 ± 1.37 ^b^
Food intake (g/day)	8.98 ± 3.65	6.76 ± 2.27	7.21 ± 3.89	7.23 ± 2.09	7.84 ± 2.69	5.85 ± 1.91
Water intake (mL/day)	22.50 ± 7.21 ^b^	33.52 ± 9.10 ^ab^	36.46 ± 7.58 ^a^	29.33 ± 7.77	25.42 ± 7.06	26.96 ± 10.15
Excreted feces (g/day)	4.70 ± 1.46 *	4.28 ± 2.23	5.32 ± 1.66 *	2.82 ± 0.92	3.01 ± 0.85	2.54 ± 1.05
Excreted urine (mL/day)	12.67 ± 7.63	15.20 ± 7.51 *	16.02 ± 6.68	7.64 ± 4.97	5.40 ± 3.17 *	10.59 ± 4.62
Carotenoids intake (μg/day)	-	20.63 ± 6.91 ^b^	55.52 ± 35.41 ^a^	-	23.92 ± 8.20 ^b^	53.24 ± 17.33 ^a^
TDF intake (g/day)	1.90 ± 0.77 *	1.65 ± 0.35 *	1.64 ± 0.18 *	0.54 ± 0.12	0.46 ± 0.12	0.42 ± 0.09
TPC intake (mg GAE/day)	19.30 ± 4.87 *	14.85 ± 3.13 *	14.26 ± 1.56 *	1.45 ± 0.42	1.41 ± 0.41	1.29 ± 0.42

^1^ Data are expressed as mean ± SD. ^a,b^ Different letters show significant statistical differences (*p* < 0.05) among groups fed the standard diet (NC, N2.5, N5) or the high fat diet (HC, H2.5, H5), after performing a one-way ANOVA. * Significant statistical difference (*p* < 0.05), after carrying out a two-sample *t* test, between the members of the NC–HC, N2.5–H2.5, and N5–H5 pairings.

**Table 3:** Biochemical parameters of plasma, inflammation and oxidative stress biomarkers analyzed in the six experimental groups at the end of the 5-week intervention period ^1^.

Parameters	NC	N2.5	N5	HC	H2.5	H5
Glucose (mg/dL)	199.4 ± 42.7 *	147.1 ± 18	132.17 ± 4.38	274.1 ± 31.1 ^a^	177.9 ± 3.89 ^b^	153.6 ± 20.5 ^b^
Proteins (g/dL)	5.42 ± 0.33 *	5.79 ± 0.18	5.67 ± 0.55	6.32 ± 0.26	6.04 ± 0.43	6.25 ± 0.12
Final ALT (U/L)	32.6 ± 5.09 *	28.95 ± 0.35 *	34.40 ± 4.16 *	47.95 ± 9.55	45.43 ± 9.83	44.30 ± 4.85
Final AST (U/L)	75.07 ± 10.14 ^b^*	88.28 ± 10.95 ^b^*	106.1 ± 6.93 ^a^*	151.10 ± 5.20	139.2 ± 43.7	142.7 ± 32.5
Adiponectin (pg/mL)	0.95 ± 0.38	0.62 ± 0.16	1.09 ± 0.84	0.55 ± 0.10	0.69 ± 0.13	0.54 ± 0.13
TNF-α (pg/mL)	15.6 ± 1.13	14.49 ± 1.29	14.09 ± 1.13	14.46 ± 1.55	14.68 ± 1.33	14.16 ± 1.62
ORAC (mmoles equiv trolox/L)	8.29 ± 1.62	9.06 ± 0.66	9.28 ± 1.17	9.79 ± 0.72	9.91 ± 1.48	9.81 ± 0.75
Urine isoprostanes (ng/mg creatinine)	0.95 ± 0.17	1.01 ± 0.18	1.1 ± 0.14	1.13 ± 0.004	0.97 ± 0.26	0.96 ± 0.09

^1^ Data are expressed as mean ± SD. ^a,b^ Different letters show significant statistical differences (*p* < 0.05) among groups fed the standard diet (NC, N2.5, N5) or the high fat diet (HC, H2.5, H5), after performing a one-way ANOVA. *Significant statistical difference (*p* < 0.05), after carrying out a two-samples *t* test, between the members of the NC-HC, N2.5-H2.5, and N5-H5 pairings. ALT: alanine aminotransferase; AST: aspartate aminotransferase; TNF: tumor necrosis factor; ORAC: oxygen radial absorption capacity.

**Table 4:** Carotenoids content (μg/g) in the liver of rats of four experimental groups, at the end of the 5-week intervention period ^1^.

Carotenoids	N2.5	N5	H2.5	H5
Lutein	nd	nd	0.03 ± 0.06	0.03 ± 0.06
α-carotene	nd	nd	0.04 ± 0.03 ^b^	0.15 ± 0.10 ^a^
β-carotene	nd	0.20 ± 0.09 ^b^	0.22 ± 0.08 ^b^	1.28 ± 0.47 ^a^
Total	nd	0.20 ± 0.09 ^b^	0.29 ± 0.12 ^b^	1.45 ± 0.51 ^a^

^1^ Data are expressed as mean ± SD. ^a,b^ Different letters shown significant statistical differences (*p* < 0.05) among groups fed the standard diet (N2.5, N5) or the high fat diet (H2.5, H5), after performing a one-way ANOVA.nd: not detected.

**Table 5:** Total fat (mg/100 g), cholesterol content (mg/g) and fatty acid concentrations (mg/g) in the liver of rats in the six experimental groups at the end of the 5-week intervention period ^1^.

Parameters	NC	N2.5	N5	HC	H2.5	H5
Total fat	4.77 ± 1.33 *	6.37 ± 0.90 *	5.41 ± 2.32 *	25.58 ± 4.98	25.09 ± 0.89	25.73 ± 0.62
Total cholesterol	223.9 ± 46 ^ab^*	275.0 ± 48 ^a^*	192.5 ± 45 ^b^	6048 ± 2801 ^a^*	647.8 ± 229 ^c^*	233.4 ± 50 ^b^
Caprylic acid (C8:0)	0.15 ± 0.03	0.15 ± 0.06	nd	0.27 ± 0.0.08 ^a^	0.13 ± 0.02 ^a,b^	0.10 ± 0.06 ^b^
Capric acid (C10:0)	0.33 ± 0.07 ^a^	0.14 ± 0.01 ^b^	0.12 ± 0.03 ^b^	0.35 ± 0.09 ^a^	0.14 ± 0.07 ^b^	0.11 ± 0.05 ^b^
Undecanoic acid (C11:0)	nd	nd	nd	0.32 ± 0.09 ^a^	0.15 ± 0.06 ^b^	nd
Lauric acid (C12:0)	0.21 ± 0.06 ^a^	0.18 ± 0.03 ^a^	0.10 ± 0.03 ^b^	0.12 ± 0.03	0.07 ± 0.02	0.07 ± 0.01
Tridecanoic acid (C13:0)	0.14 ± 0.05 ^a^	0.11 ± 0.03 ^a^	0.06 ± 0.007 ^b^	0.14 ± 0.01 ^a^	0.12 ± 0.02 ^a^	0.09 ± 0.03 ^b^
Myristic acid (C14:0)	0.66 ± 0.34 ^a^	0.18 ± 0.03 ^b^	0.13 ± 0.02 ^b^	1.67 ± 0.08 ^a^	1.28 ± 0.16 ^b^	1.29 ± 0.27 ^b^
Pentadecanoic acid (C15:0)	0.21 ± 0.10 ^a^	0.08 ± 0.008 ^b^	0.08 ± 0.009 ^b^	0.37 ± 0.02	0.36 ± 0.05	0.34 ± 0.01
Palmitic acid (C16:0)	7.79 ± 0.41 ^a^	7.83 ± 0.98 ^ab^	6.78 ± 0.85 ^b^	22.63 ± 2.98	20.46 ± 2.17	19.13 ± 1.46
Margaric acid (C17:0)	0.39 ± 0.21 ^a^	0.16 ± 0.03 ^b^	0.16 ± 0.02 ^b^	0.46 ± 0.006 ^a^	0.36 ± 0.04 ^b^	0.36 ± 0.03 ^b^
Stearic acid (C18:0)	5.00 ± 0.42	4.42 ± 0.70	4.95 ± 0.81	8.08 ± 0.23 ^a^	6.35 ± 0.88 ^b^	6.73 ± 0.88 ^b^
Arachidic acid (C20:0)	0.80 ± 0.13 ^a^	0.10 ± 0.02 ^b^	0.10 ± 0.03 ^b^	0.40 ± 0.13 ^a^	0.25 ± 0.16 ^ab^	0.13 ± 0.02 ^b^
Myristoleic acid (C14:1)	0.08 ± 0.01	0.10 ± 0.04	0.10 ± 0.03	0.12 ± 0.03	0.11 ± 0.08	0.12 ± 0.07
Cis Pentadecanoic acid (C15:1)	0.16 ± 0.07 ^b^	0.18 ± 0.06 ^b^	0.35 ± 0.05 ^a^	0.10 ± 0.03	0.11 ± 0.04	0.11 ± 0.03
Palmitoleic acid (C16:1)	0.70 ± 0.04 ^a^	0.51 ± 0.19 ^b^	0.51 ± 0.09 ^b^	2.98 ± 0.64	3.63 ± 0.70	3.63 ± 0.68
Cis Heptadecenoic acid (C17:1)	0.07 ± 0.03	0.06 ± 0.02	0.06 ± 0.009	0.32 ± 0.05	0.39 ± 0.07	0.43 ± 0.09
Oleic cid (C18:1n9c)	3.27 ± 0.11	3.47 ± 0.24	3.25 ± 0.41	30.99 ± 2.68	39.58 ± 5.81	40.94 ± 8.92
Eicosenoic acid (C20:1n9)	0.06 ± 0.01	0.06 ± 0.02	0.08 ± 0.04	0.31 ± 0.02	0.38 ± 0.06	0.36 ± 0.09
Nervonic acid (C24:1n9)	0.33 ± 0.03 ^b^	0.39 ± 0.08 ^b^	0.55 ± 0.09 ^a^	0.32 ± 0.04 ^a^	0.46 ± 0.10 ^ab^	0.58 ± 0.13 ^b^
Linolelaidic acid (C18:2tn-6)	nd	nd	nd	nd	0.11 ± 0.04	0.11 ± 0.02
Linoleic acid (C18:2cn-6)	5.36 ± 0.85 ^b^	6.97 ± 1.20 ^a^	6.58 ± 0.61 ^a^	13.87 ± 1.28	14.44 ± 1.73	18.05 ± 4.21
γ-Linolenic acid (C18:3n-6)	0.21 ± 0.10 ^a^	0.08 ± 0.02 ^b^	0.06 ± 0.03 ^b^	0.14 ± 0.04	0.17 ± 0.05	0.18 ± 0.04
α- Linolenic acid (C18:3n-3)	0.32 ± 0.09 ^a^	0.18 ± 0.07 ^b^	0.28 ± 0.05 ^ab^	0.51 ± 0.06 ^b^	1.19 ± 0.27 ^a^	1.39 ± 0.3 ^a^
Eicosadienoic acid (C20:2n-6)	0.11 ± 0.05 ^b^	0.17 ± 0.04 ^a^	0.15 ± 0.03 ^ab^	0.28 ± 0.11 ^b^	0.43 ± 0.07 ^a^	0.42 ± 0.08 ^a^
Dihomo-γ-linolenic acid (C20:3n-6)	0.17 ± 0.01 ^b^	0.19 ± 0.03 ^b^	0.32 ± 0.07 ^a^	0.73 ± 0.06	0.84 ± 0.23	0.81 ± 0.06
Arachidonic acid (C20:4n-6)	5.76 ± 0.82	5.81 ± 0.61	6.27 ± 0.97	4.40 ± 0.42	5.00 ± 1.04	5.40 ± 0.70
Eicosapentaenoic acid (C20:5n-3)	nd	nd	0.19 ± 0.03	nd	0.14 ± 0.09 ^b^	0.28 ± 0.05 ^a^
Docosahexaenoic acid (C22:6n-3)	1.19 ± 0.13 ^b^	1.37 ± 0.01 ^a^	1.53 ± 0.16 ^a^	0.84 ± 0.10 ^b^	1.04 ± 0.23 ^ab^	1.28 ± 0.16 ^a^

^1^ Data are expressed as mean ± SD. ^a–c^ Different letters show significant statistical differences (*p* < 0.05) among groups fed the standard diet (NC, N2.5, N5) or the high fat diet (HC, H2.5, H5), after performing a one-way ANOVA. nd: Not detected. * Significant statistical difference (*p* < 0.05), after carrying out a two-sample *t* test, between the members of the NC-HC, N2.5-H2.5, and N5-H5 pairings.

**Table 6:** Gene symbol, gene name, and relative fold change of the genes that showed an over or down-expression value higher than 2 (*p* < 0.05) in the rat livers ^1^.

Symbol	Gene Name	NC-HC	NC-N5	NC-H5
β-oxidation				
*ACADL*	Acyl-coenzyme A dehydrogenase, long-chain	-	3.03	-
*CPT1A*	Carnitine palmitoyltransferase 1A, liver	-	-	2.25
*CPT2*	Carnitine palmitoyltransferase 2	-	7.97	2.24
*PPARA*	Peroxisome proliferator activated receptor alpha	-	2.75	2.44
Cholesterol transport and metabolism				
*ABCG1*	ATP-binding cassette, subfamily G (WHITE), member 1	5.89	2.26	-
*APOA1*	Apolipoprotein A-I	-	7.86	-
*APOB*	Apolipoprotein B	-	2.68	-
*APOE*	Apolipoprotein E	-	36.06	3.17
*CNBP*	CCHC-type zinc finger, nucleic acid binding protein	-	4.70	-
*CYP2E1*	Cytochrome P450, family 2, subfamily E, polypeptide 1	-	2.94	2.95
*CYP7A1*	Cytochrome P450, family 7, subfamily A, polypeptide 1	-	-	2.47
*LDLR*	Low density lipoprotein receptor	-	4.76	-
*NR1H3*	Nuclear receptor subfamily 1, group H, member 3	-	2.23	-
*NR1H4*	Nuclear receptor subfamily 1, group H, member 4	-	11.29	2.88
*PPARD*	Peroxisome proliferator-activated receptor delta	-	2.93	-
*PPARG*	Peroxisome proliferator-activated receptor gamma	3.36	6.81	3.85
*SREBF2*	Sterol regulatory element binding transcription factor 2	-	3.66	-
Other lipid transport and metabolism				
*ACSM3*	Acyl-CoA synthetase medium-chain family member 3	-	2.27	-
*LPL*	Lipoprotein lipase	2.98	-	-
Inflammatory response and apoptosis				
*ADIPOR1*	Adiponectin receptor 1	-	6.96	2.63
*FAS*	Fas (TNF receptor superfamily, member 6)	2.15	-	-
*IL1B*	Interleukin 1 beta	7.04	3.23	-
*NFKB1*	Nuclear factor of kappa light polypeptide gene enhancer in B-cells 1	-	3.05	-
*CASP3*	Caspase 3	-	2.62	3.02
*MAPK8*	Mitogen-activated protein kinase 8	-	2.77	-
*SOCS3*	Suppressor of cytokine signaling 3	-	5.77	-

^1^ The fold change for each gene in groups N5, H5, and HC was calculated taking as reference a value of 1 for the group NC. ATP: adenosine triphosphate.

**Table 7:** Redox ratios in the liver of the rats of four experimental groups at the end of the intervention period ^1^.

Ratio	NC	N5	HC	H5
GSH/GSSG	1.39 ± 0.38 *	0.79 ± 0.16	0.97 ± 0.30	0.97 ± 0.21
NAD/NADH	0.33 ± 0.09	0.31 ± 0.06	0.36 ± 0.04	0.36 ± 0.10
NADP/NADPH	1.0 ± 0.26	1.29 ± 0.49	1.27 ± 0.59	1.27 ± 0.23

^1^ Data are expressed as mean ± SD. * Significant statistical difference (*p* < 0.05), after carrying out a two-sample *t* test comparing the NC–N5 and HC–H5 groups.

## Abbreviations

GC	Gas chromatography
HPLC-MS	High-performance liquid chromatography–mass spectrometry
DAD	Diode array detector
PCR	Polymerase chain reaction
Asp	l-aspartic acid
Ala	l-alanine
Ser	l-serine
Pro	l-proline
Val	l-valine
Thr	l-threonine
Cys	l-cysteine
Glu	l-glutamic acid
Ile/Leu/OH-Pro	l-isoleucine/l-leucine/l-hydroxyproline
Met	l-methionine
Gly	Glycine
Homo-Cys	l-homocysteine
Lys/Gln	l-lysine/ l-glutamine
Asn	l-asparagine
His	l-histidine
Phe	l-phenylalanine
Arg	l-arginine
Tyr	l-tyrosine
Trp	l-tryptophan
GSH	l-glutathione
GSSG	l-glutathione oxidised form
NAD	Nicotinamide adenine dinucleotide oxidised form
NADH	Nicotinamide adenine dinucleotide reduced form
NADP	Nicotinamide adenine dinucleotide phosphate oxidised form
NADPH	Nicotinamide adenine dinucleotide phosphate reduced form
Hypoxan	Hypoxanthine
CMP	Cytidine monophosphate
AMP	Adenosine monophosphate
GMP	Guanosine monophosphate
UDP	Uridine diphosphate
GDP	Guanosine diphosphate
CTP	Cytidine triphosphate
ATP	Adenosine triphosphate
ITP	Inosine triphosphate
